# Geranylgeraniol Application in Human Osteoblasts and Osteoclasts for Reversal of the Effect of Bisphosphonates

**DOI:** 10.3390/life13061353

**Published:** 2023-06-08

**Authors:** Junho Jung, Jung Soo Park, Jeewan Chun, Bilal Al-Nawas, Thomas Ziebart, Yong-Dae Kwon

**Affiliations:** 1 Department of Oral & Maxillofacial Surgery, School of Dentistry, Kyung Hee University, Seoul 02447, Republic of Korea; 2 Department of Periodontology, Korea University Anam Hospital, Seoul 02841, Republic of Korea; 3 Department of Dentistry, Graduate School, Kyung Hee University, Seoul 02447, Republic of Korea; 4 Department of Oral and Maxillofacial Surgery, University Medical Center of the Johannes Gutenberg University Mainz, 55131 Mainz, Germany; 5 Department of Oral and Maxillofacial Surgery, University Hospital Giessen and Marburg of the Philipps University of Marburg, 35043 Marburg, Germany

**Keywords:** geranylgeraniol, GGOH, bisphosphonates, medication-related osteonecrosis of the jaw, MRONJ, mevalonate pathway

## Abstract

Nitrogen-containing bisphosphonates lead to the depletion of geranylgeranyl pyrophosphate involved in the mevalonate pathway. The effect of geranylgeraniol (GGOH) on human osteoblast and osteoclast activities suppressed by zoledronate was investigated in this study. The effect of GGOH on human osteoblasts and osteoclasts subjected to treatment with zoledronate was analyzed by assessing cell viability, osteoclast differentiation, resorption ability, gene expression, and protein synthesis. Cell viability suppressed by bisphosphonates in osteoblasts and osteoprogenitor cells was restored with GGOH. Osteoclast differentiation was analyzed by vitronectin receptor immunofluorescence staining, and the addition of GGOH to zoledronate significantly increased osteoclast differentiation compared with zoledronate alone. A trend of reversal of osteoclast resorption by GGOH was observed; however, it was not significant in all groups. The expression of ALP, type 1 collagen, and RUNX2 in osteoblasts was recovered by the addition of GGOH. Only CALCR expression in osteoclasts was significantly recovered by GGOH addition in the zoledronate group. Although the activities of osteoblasts and osteoclasts were not entirely restored, the possibility that the topical application of GGOH in MRONJ patients or patients with dental problems and bisphosphonates might lessen the risk of development and recurrence of MRONJ is shown.

## 1. Introduction

Bisphosphonates are pharmacological agents that have been used for over 40 years for the treatment of bone diseases, such as osteoporosis, Paget’s disease, osteogenesis imperfecta, multiple myeloma, fibrous dysplasia, and bone metastasis mainly caused by prostate and breast cancer [1,2,3,4,5,6,7]. However, medication-related osteonecrosis of the jaw (MRONJ) was first described in 2003, and several cases of osteonecrosis with a history of administration of bisphosphonates have been reported [8,9]. Considering their efficacy and cost-effectiveness, it is not surprising that bisphosphonates continue to be broadly used for the prevention and treatment of such diseases, despite the exhibition and development of various complications [10,11,12]. As the number of elderly individuals increases, the prevalence of bone diseases also increases, and the prescription of bisphosphonates and the complications resulting from their use are expected to remain high [13].

Nitrogen-containing bisphosphonates inhibit the actions of farnesyl pyrophosphate synthase, an enzyme involved in the mevalonate pathway, leading to the depletion of geranylgeranyl pyrophosphate (GGPP), an intermediate in the mevalonate pathway [14,15]. GGPP is an essential substrate for post-translational prenylation of small GTP-binding proteins, such as Rab and Rho family proteins [16,17]. Since geranylgeraniol (GGOH) serves as a substrate for GGPP formation, supplementation of GGOH may help resume prenylation of small GTP-binding proteins that play a key role in cell survival, despite the presence of bisphosphonates [18,19]. Therefore, several studies have noticed and focused on the possibility of rescuing cells by using this strategy [18,19,20,21,22].

Although clear standards for treatment of MRONJ have not been determined thus far, early surgical interventions are gaining preference among oral and maxillofacial surgeons, as higher success rates for the management of MRONJ have been reported [23]. However, there is a possibility that surgical treatment may act as a trigger for MRONJ development. Therefore, adjunctive therapies, including the administration of teriparatide, pentoxifylline, and tocopherol to reduce surgical morbidity, have been suggested [24,25]. As described above, if GGOH can aid the recovery of the cellular damage caused by bisphosphonates, it may emerge as a potential candidate for the treatment of MRONJ. Additionally, the use of GGOH as a preventive measure can also be considered. The effectiveness of GGOH in rescuing and preventing cellular damage has been recently presented [26,27],

Thus, this study aimed to investigate the effect of GGOH on human osteoblast and osteoclast activities, including cell proliferation, osteoclastogenesis, resorption ability, gene expression, and protein synthesis, in the presence of various bisphosphonates.

## 2. Materials and Methods

### 2.1. Cell Culture

Commercially available human osteoblast cells (PromoCell) and human osteoclast precursors (Lonza) were cultured in their respective culture media (PromoCell and Lonza) at 37 °C with 5% CO_2_, and the media were replaced with fresh media every three days. The osteoblasts were passaged when they reached 70–80% confluence in a 75 cm^2^ culture flask (Corning, New York, NY, USA) using Accutase solution (Sigma-Aldrich, St. Louis, MO, USA). Osteoblasts from passages 4 to 7 were used in the experiments and seeded onto appropriate plates at a density of 2 × 10^4^ cells/cm^2^. The osteoclast precursors were seeded onto appropriate plates for each experiment at a density of 4 × 10^4^ cells/cm^2^, and human receptor activators of nuclear factor kappa-B ligand (RANKL; 40 ng/mL, Sigma-Aldrich) and human macrophage colony stimulating factor (M-CSF; 25 ng/mL, Sigma-Aldrich) were added to induce osteoclast differentiation.

### 2.2. Bisphosphonates and Geranylgeraniol

Nitrogen-containing bisphosphonates, namely, Zometa (zoledronate, Novartis Pharma, Basel, Switzerland) and Alendron (alendronate, Hexal AG; Novartis group, Holzkirchen, Germany), and a non-nitrogen-containing bisphosphonate, Bonefos (clodronate, Bayer, Leverkusen, Germany), were used for the experiments, and geranylgeraniol (GGOH) purchased from Sigma-Aldrich was supplemented along with bisphosphonate treatment. The alendronate tablet was pulverized and dissolved in phosphate-buffered saline (PBS). Subsequently, the pH of the solution was adjusted, and the solution was sterilized by filtration.

### 2.3. Cell Viability

The viability of human osteoblasts and human osteoclast precursors was determined using the MTT colorimetric assay (Sigma-Aldrich). Osteoblasts and osteoclast precursor cells were seeded onto 24-well plates at a density of 2 × 10^4^ cells/cm^2^ and incubated for 24 h. Bisphosphonates, namely, zoledronate (Zol), alendronate (Aln), and clodronate (Clod), were added to the wells (0, 10, and 50 μM) with or without GGOH (10 μM). The above-mentioned range of concentrations was selected, because in vivo concentrations in plasma shortly after zoledronate infusion are approximately 5 μM, and these doses roughly correspond to the standard oncology dose regimens of 1 year and 4 years (32, 33). The assay was conducted in triplicate, with three replicates for each experiment. After incubation for a duration of 72 h, MTT solution was added to the wells and the plates were incubated for 4 h. Cells were subjected to lysis for 30 min using a lysis buffer (isopropanol and 2 N HCl, 49:1), and the optical density (OD) was measured at 570 nm using a spectrophotometric microplate reader (Synergy HT; BioTek, Winooski, VT, USA).

### 2.4. Vitronectin Receptor Staining and Cell Count

To analyze osteoclast differentiation, vitronectin receptor (VNR) immunofluorescence staining was performed. VNR-positive multinucleated cells (cells with more than 2 nuclei) were considered osteoclasts. The osteoclast precursors were seeded onto 96-well plates at a density of 4 × 10^4^ cells/cm^2^, and human receptor activators of nuclear factor kappa-B ligand (RANKL; 40 ng/mL, Sigma-Aldrich) and human macrophage colony stimulating factor (M-CSF; 25 ng/mL, Sigma-Aldrich) were added. The assay was conducted in triplicate. After incubation for a duration of 24 h, bisphosphonates (50 μM) with or without GGOH (10 μM) were added and the plates were incubated for 72 h. Subsequently, the cells were subjected to fixation in 3.7% paraformaldehyde for 20 min at 25 °C and blocked with goat serum (Thermo Fisher Scientific, Waltham, MA, USA) for 30 min at 25 °C. The cells were then incubated with anti-human CD51/61 antibody (MAB1976Z, 1:200; EMD Millipore, Temecula, CA, USA) for 1 h at 25 °C and with a secondary fluorescent antibody (ab150113, 1:500; Abcam, Cambridge, United Kingdom) for 1 h at 25 °C. Images were obtained using a fluorescence microscope (BZ-9000; Keyence, Osaka, Japan) and subsequently merged with the DAPI images for nuclei staining. For each group, fifteen different areas, each measuring 0.01 cm^2^, were selected, and a cell count was performed using ImageJ software. The proportion of cells with more than two nuclei among the total cell population was calculated.

### 2.5. Bone Resorption Pit Assay

After incubation of osteoclast precursor cells with M-CSF and RANKL for 72 h as per the methods described above, the cells were counted and seeded onto 24-well hydroxyapatite-coated plates (Corning) at a density of 2 × 10^4^ cells/cm^2^. The assay was conducted in quadruplicate with two replicates for each experiment. After incubation for a duration of 24 h, bisphosphonates (10 and 50 μM) with or without GGOH (10 μM) were added. Following incubation for 72 h, resorption pits were observed using an inverted phase-contrast microscope and analyzed by measuring the resorbed area using ImageJ software.

### 2.6. Messenger RNA Extraction and Quantitative Real-Time Polymerase Chain Reaction

Osteoblasts were seeded onto 6-well plates and incubated with Aln and Zol (50 μM) with or without GGOH (10 μM) for 72 h. Osteoclast precursors were also seeded onto 6-well plates and differentiated into osteoclasts by RANKL and M-CSF stimulation. Thereafter, Aln and Zol, with or without GGOH, were added for the conduction of treatment for 72 h. Subsequently, the cells were harvested using Accutase solution, and mRNA extraction was performed. The amount of extracted mRNA was measured spectrophotometrically at 260 nm. Complementary DNA synthesis was performed using a cDNA synthesis kit (iScrip; Bio-Rad, Hilden, Germany) and a thermal cycler (peqSTAR 96 Universal Gradient; PeqLab, Erlangen, Germany). The sequences of primers used in the experiment are listed in Table 1. iQ SYBR Green Supermix (Bio-Rad) was used to conduct polymerase chain reactions (PCRs). The PCR cycling conditions used in the present study were as follows: initial denaturation at 95 °C for 5 min, 40 cycles of denaturation at 95 °C for 30 s, annealing at 56 °C for 30 s, and elongation at 71 °C for 20 s, with final elongation at 65 °C for 0.5 s and then at 95 °C for 5 s (CFX Connect; Bio-Rad). Amplification of PCR products was confirmed by performing a melting curve analysis. The expression levels of target genes were normalized using glyceraldehyde 3-phosphate dehydrogenase (GAPDH) and were quantified using the ΔΔCT method [28]. The assay was conducted in triplicate, with three replicates for each experiment.

### 2.7. Western Blot Analysis

Osteoblasts were incubated with zoledronate (50 μM) for 72 h. Subsequently, the cells were subjected to lysis with RIPA buffer (Sigma-Aldrich) containing a phosphatase inhibitor cocktail (Sigma-Aldrich). Protein concentration was determined using a BCA protein assay kit. Sodium dodecyl sulfate-polyacrylamide gel electrophoresis (SDS-PAGE) was used to separate proteins, which were then transferred onto a PVDF membrane, followed by blocking for 1 h. The membrane was then incubated for 24 h at 4 °C with primary antibodies against the following molecules: alkaline phosphatase (ALP), macrophage colony-stimulating factor (M-CSF), receptor activator of nuclear factor kappa-B ligand (RANKL), and type 1 collagen (cat. nos. ab108337, ab52864, ab9957, and ab138492, respectively; dilutions of 1:10,000, 1:50,000, 1 μg/mL, and 1:1000, respectively; Abcam), RUNX2 (cat no. 12556, dilution 1:1000, Cell Signaling Technology, Danvers, MA, USA), β-actin (cat. no. sc-47778, dilution 1:3000, Santa Cruz Biotechnology, Dallas, TX, USA). Subsequently, they were incubated with anti-mouse and anti-rabbit secondary horseradish peroxidase-conjugated antibodies (cat. nos. 111-035-003 and 115-035-003, both with dilutions of 1:5000; Jackson ImmunoResearch Laboratories, West Grove, PA, USA) for 1 h at room temperature. The immunoreactive proteins were determined using enhanced ECL chemiluminescence and a detection device (Fusion Solo S; Vilber, Marne-la-Vallée, France).

### 2.8. Statistical Analysis

The Kruskal–Wallis test and the Mann–Whitney *U* test with Bonferroni correction were used to perform statistical analyses. Two-way ANOVA with Dunnett’s T3 test was used to analyze the osteoclast differentiation count, as the normality of data was proven. All statistical analyses were performed using SPSS (version 25.0; IBM Corp). Statistical significance was set at *p* < 0.05.

## 3. Results

### 3.1. Cell Viability

**Figure 1:**
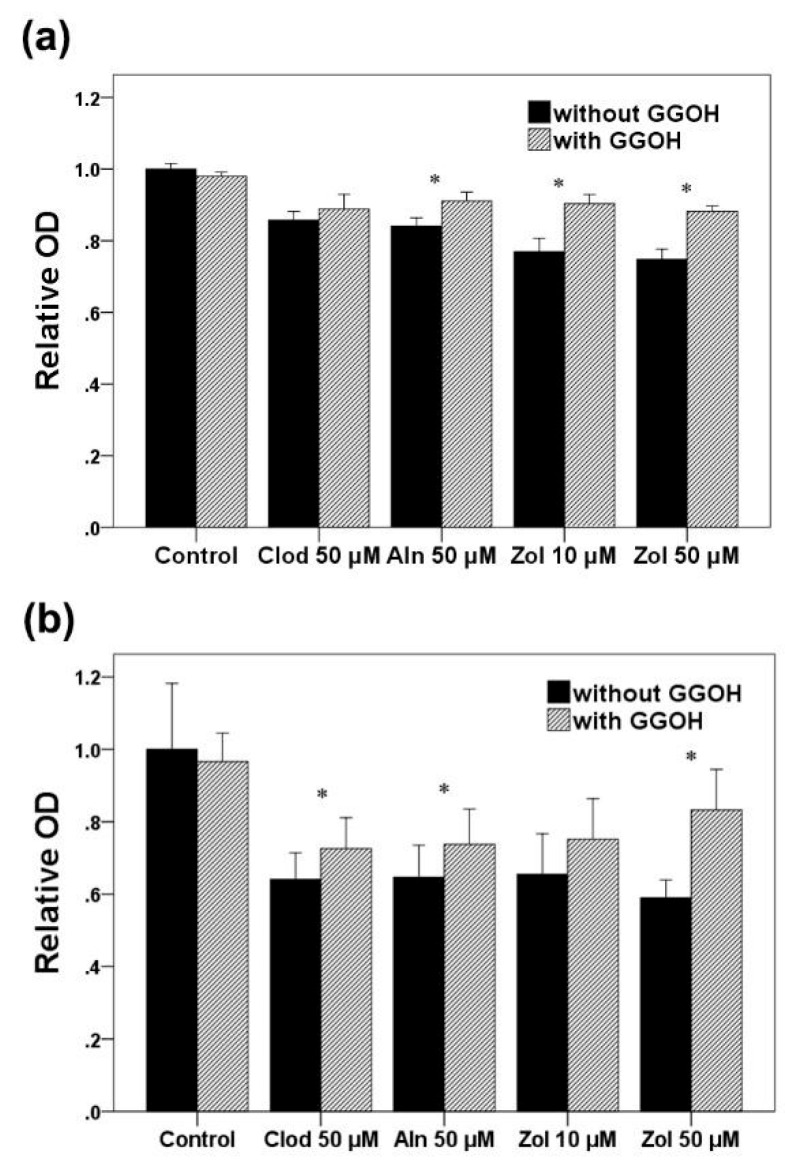
Cell viability assessed in osteoblasts (**a**) and osteoclast precursors (**b**). The values have been normalized relative to the control. Asterisks (*) represent statistical significance for comparisons of the groups with GGOH and without GGOH addition (*p* < 0.05), and the error bars indicate standard deviations. Clod, clodronate; Aln, alendronate; Zol, zoledronate; GGOH, geranylgeraniol.

Based on the statistical analysis results, a significant difference was observed between the groups in osteoblasts (Figure 1). Bisphosphonate treatment decreased cell viability compared to that observed in the control group (*p* < 0.001). The Zol 50 μM group showed the most considerable decrease among the bisphosphonate-treated groups. The addition of GGOH demonstrated a statistically significant improvement in cell viability in the Aln and Zol groups (*p* = 0.038 and 0.002, respectively).

Regarding osteoclast progenitors, a significant difference was observed between the groups (*p* < 0.001). Zol 50 μM was determined as the most potent inhibitor of cell viability. Cell viability was significantly improved by GGOH supplementation (*p* < 0.001), except in the Zol 10 μM group (*p* = 0.089).

### 3.2. Osteoclast Differentiation

**Figure 2:**
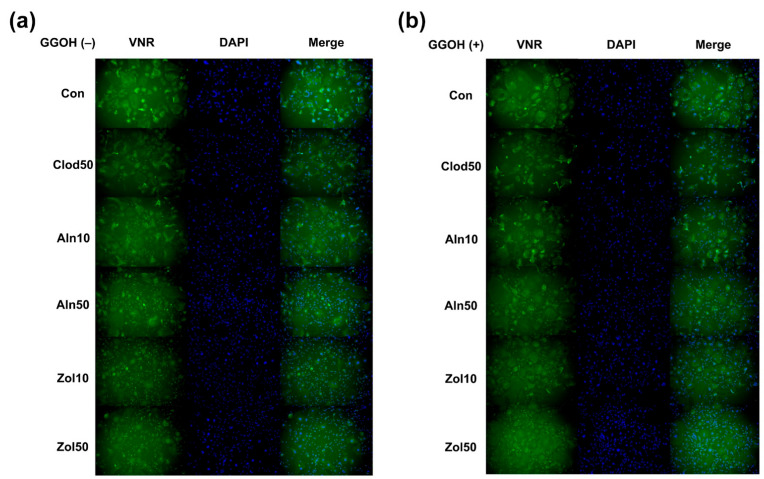
Vitronectin receptor (VNR) immunofluorescence staining conducted for cells with GGOH (**a**) and without GGOH addition (**b**). VNR-positive multinucleated cells (cells with more than two nuclei) were considered osteoclasts. Clod, clodronate; Aln, alendronate; Zol, zoledronate; GGOH, geranylgeraniol.

**Figure 3:**
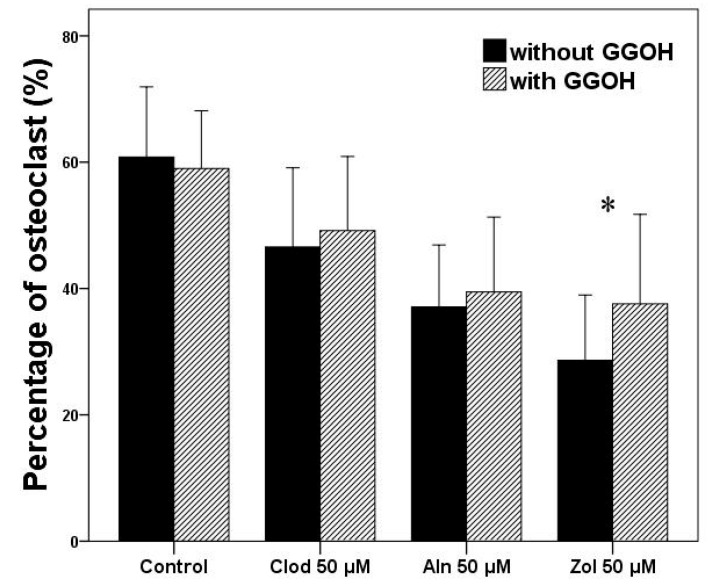
VNR-positive multinucleated cell counts. The percentage of multinucleated cell (cells with more than two nuclei) was determined with respect to the total cell number. Asterisks (*) represent statistical significance for comparisons of the groups with GGOH and without GGOH addition (*p* < 0.05), and the error bars indicate standard deviations. Clod, clodronate; Aln, alendronate; Zol, zoledronate; GGOH, geranylgeraniol.

Osteoclast differentiation was evaluated by counting the number of cells with more than two nuclei, showing positive results for VNR, and by calculating the proportion of such cells among the total cell population (Figure 2 and Figure 3). A statistically significant difference was observed between the groups (*p* = 0.019). The number of osteoclasts decreased with bisphosphonate treatment, and Zol showed the most considerable potency among bisphosphonates, followed by Aln and Clod. The addition of GGOH to Zol significantly increased osteoclast differentiation compared to that observed with Zol alone (*p* = 0.027). In the Clod and Aln groups, although the differentiation increased with GGOH, the increase was not found to be statistically significant.

### 3.3. Resorption Area Analysis

**Figure 4:**
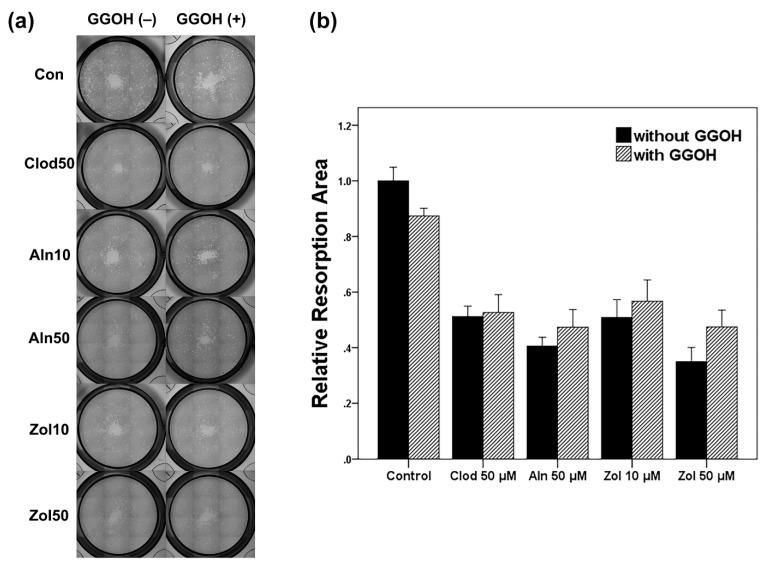
(**a**) Resorption pit assay conducted to analyze the resorption ability of osteoclast and osteoclast differentiation. (**b**) The resorbed area measured was normalized relative to the control. Asterisks (*) represent statistical significance for comparisons of the groups with GGOH and without GGOH addition (*p* < 0.05), and the error bars indicate standard deviations. Clod, clodronate; Aln, alendronate; Zol, zoledronate; GGOH, geranylgeraniol.

To analyze the resorption ability of osteoclasts and the extent of osteoclast differentiation, a resorption pit assay was conducted (Figure 4). A significant difference in resorption area was observed between the groups. The resorption area decreased with the addition of bisphosphonates. The most considerable decrease in resorption was observed in the Zol 50 μM group (*p* < 0.001). Although a trend of reversal of the resorption of osteoclasts by GGOH was observed, it was not statistically significant in all groups (Control, Clod, Aln, and Zol groups; *p* = 0.094, *p* = 0.574, *p* = 0.645, and *p* = 0.072, respectively).

### 3.4. Gene Expression and Protein Synthesis

#### 3.4.1. Osteoblasts

**Figure 5:**
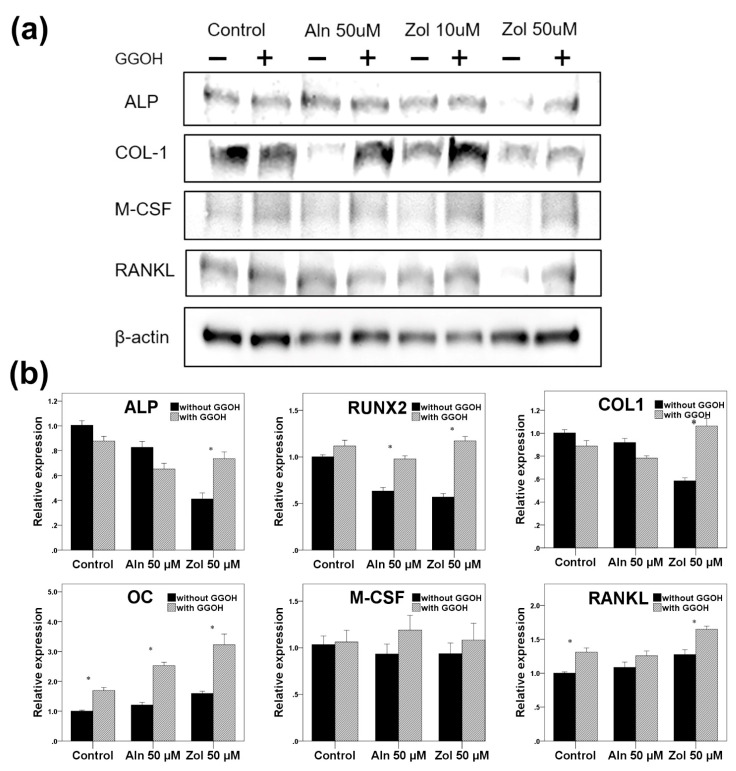
(**a**) Western blot analysis and (**b**) qRT-PCR conducted for osteoblasts. The gene expression levels have been normalized relative to the control. Asterisks (*) represent statistical significance for comparisons of the groups with GGOH and without GGOH addition (*p* < 0.05), and the error bars indicate standard deviations. ALP, alkaline phosphatase; COL1, type 1 collagen; OC, osteocalcin; M-CSF, macrophage colony stimulating factor; RANKL, receptor activator of nuclear factor kappa-B ligand; Aln, alendronate; Zol, zoledronate; GGOH, geranylgeraniol.

qRT-PCR was performed to analyze the gene expression of the osteogenic markers and osteoclastogenesis-stimulating genes, including ALP, RUNX2, type 1 collagen, osteocalcin, M-CSF, and RANKL (Figure 5).

The gene expression of ALP, type 1 collagen, and RUNX2 in osteoblasts significantly decreased as bisphosphonates were administered compared to the control group (*p* < 0.001). The addition of GGOH reversed the effects of bisphosphonates on the expression of the above-mentioned genes (*p* < 0.001). Although no statistical significance was observed in the gene expression of M-CSF, bisphosphonates decreased the expression and GGOH reversed the action of Zol (*p* = 0.836). RANKL and osteocalcin gene expression levels increased with bisphosphonate treatment, and GGOH significantly enhanced the expression (*p* = 0.001).

Western blot analysis (Figure 5) showed that incubation with Zol (50 μM) reduced the expression of ALP, type 1 collagen, M-CSF, and RANKL compared to the control. ALP, M-CSF, and RANKL expression demonstrated recovery with the addition of GGOH to the osteoblasts.

#### 3.4.2. Osteoclasts

**Figure 6:**
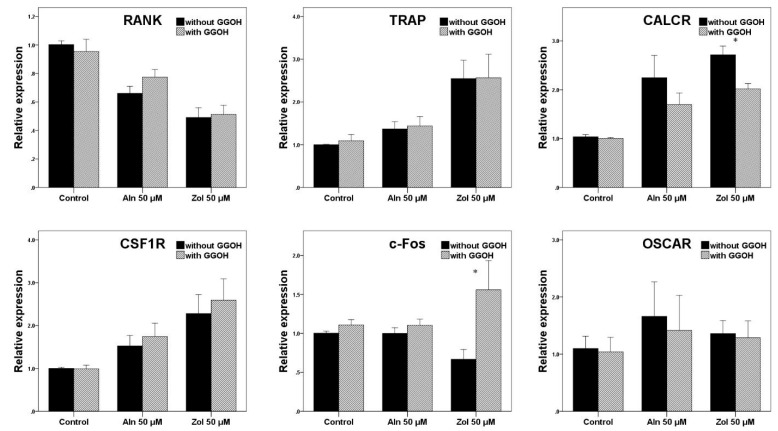
qRT-PCR analysis conducted for osteoclasts. The gene expression levels have been normalized relative to the control. Asterisks (*) represent statistical significance for comparisons of the groups with GGOH and without GGOH addition (*p* < 0.05), and the error bars indicate standard deviations. RANK, receptor activator of nuclear factor kappa-B; TRAP, tartrate-resistant acid phosphatase; CALCR, calcitonin receptor; CSF1R, colony stimulating factor 1 receptor; c-Fos, OSCAR, osteoclast-associated receptor; Aln, alendronate; Zol, zoledronate; GGOH, geranylgeraniol.

qRT-PCR was performed to analyze the expression of genes related to osteoclast differentiation and activity, including receptor activator of nuclear factor kappa-B (RANK), TRAP, calcitonin receptor (CALCR), colony stimulating factor 1 receptor (CSF1R), c-Fos, and osteoclast-associated receptor (OSCAR) (Figure 6).

Bisphosphonate treatment significantly increased the gene expression of TRAP, CALCR, CSF1R, and OSCAR compared to the control group. However, only CALCR expression was significantly recovered by GGOH in the Zol group (*p* = 0.011). In the case of TRAP and CSF1R, GGOH treatment did not cause a significant difference in expression (*p* = 0.730 and *p* = 0.258, respectively). The gene expression of RANK and c-Fos in the bisphosphonate-treated group was significantly decreased compared to that observed in the control. The effect of GGOH increased the gene expression of RANK; however, the difference was not statistically significant (*p* = 0.895). GGOH had a significant effect on c-Fos expression after Zol treatment (*p* = 0.030).

## 4. Discussion

Almost 20 years have elapsed since the first documentation of MRONJ, and it has emerged as a disease well recognized by clinicians [29]. Several attempts to unearth the nature of the disease have been reported; however, an established pathophysiology or treatment regimen remain unavailable. Several hypotheses regarding the cause of the disease have been suggested. Impaired bone turnover, exertion of cell toxicity against various cell types, bacterial infection, and inflammation occurring due to dental problems and microtrauma have been considered as contributing factors. MRONJ is therefore considered a multifactorial disease [30]. Among these possible causes, impaired bone turnover occurring due to downregulated osteoclast and osteoblast activities plays a critical role in the development of MRONJ.

MRONJ was first reported in patients subjected to treatments using nitrogen-containing bisphosphonates, and such bisphosphonates are broadly prescribed as antiresorptive agents for the treatment of various bone diseases. Nitrogen-containing bisphosphonates inhibit the actions of farnesyl pyrophosphate synthase (FPPS), an enzyme involved in the mevalonate pathway, leading to the depletion of geranylgeranyl pyrophosphate (GGPP), an essential substrate for the post-translational prenylation of small GTP-binding proteins, such as those belonging to Rab and Rho subfamilies [14,15]. The prenylated small GTP-binding proteins act as molecular switches during signal transduction and help regulate cellular processes and functions, including cell growth and survival, differentiation and proliferation, gene expression, and energy metabolism [31,32]. Thus, undermining of the function and viability of osteoclasts and osteoblasts by nitrogen-containing bisphosphonates leads to a reduction in the extent of bone remodeling, and the use of nitrogen-containing bisphosphonates has been ascertained as the main cause of MRONJ.

Certain researchers have focused on the application of geranylgeraniol (GGOH) as a potential therapeutic approach, as it serves as a substrate for GGPP and undergoes conversion to GGPP [18]. A study conducted by Ziebart et al. reported that the addition of GGOH reversed the effect of nitrogen-containing bisphosphonates, resulting in increased cell viability and migration and decreased cell apoptosis [18]. Another study also reported that the cytotoxic effect of Zol on osteoblasts was reversed by GGOH addition [33]. Furthermore, the prenylation of Rap1A, a GTP-binding protein, was restored by GGOH addition in the presence of bisphosphonates exerting effects on human osteoclasts [27].

Decreased osteoclasts and their suppressed resorption ability under bisphosphonates have a critical effect on the occurrence of MRONJ due to impaired bone turnover. However, they have not been investigated in human osteoclasts, despite the importance of doing so. The present study focused on analyzing human osteoclast differentiation and activities, and the results showed positive effects of GGOH exerted on human osteoblasts and osteoclasts subjected to treatment with Zol. Notably, osteoclast differentiation compromised by Zol treatment was restored to a certain extent, although this level was not sufficient to reach values demonstrated by the control. Given that the expression levels of M-CSF and RANKL reduced by BPS in osteoblasts were also recovered by GGOH, it is assumed that it has the potential to not only promote increased bone formation but also enhance osteoclastogenesis. The addition of GGOH also tends to improve hydroxyapatite resorption. Given that the expression levels of M-CSF and RANKL reduced by BPS in osteoblasts were also recovered by GGOH, it is assumed that it has the potential to not only promote increased bone formation but also enhance osteoclastogenesis and bone resorption in vivo. Gene expression, such as ALP, collagen 1, RANKL, and RUNX2 expression in osteoblasts and C-Fos and CALCR expression in osteoclasts, was significantly recovered with the addition of GGOH. Interestingly, not all genes and proteins were affected. Since protein prenylation is mediated not only by the action of geranylgeranyl transferase, but also by the action of farnesyltransferase for farnesylation of proteins [32,34], the sole addition of GGOH may be insufficient to fully restore cellular function, and as a result the gene expression and protein expression were not correlated accordingly.

Aln and Clod also exhibited suppressive effects on cell survival, osteoclastogenesis, resorption, and gene expression. As Clod is not a nitrogen-containing BP that acts via the mevalonate pathway, it is not surprising that GGOH has no impact on cells treated with Clod. However, in the case of Aln, the effect of GGOH was also not profound. Although 10 μM of GGOH increased the viability of osteoclast precursors treated with Aln, similar to Zol, there was no statistically significant impact on osteoclast differentiation. Considering that the drug potency of Aln is lower than that of Zol, resulting in a lesser effect on bone turnover, higher concentrations of Aln could be comparable to Zol, and supplementation with varying amounts of GGOH might lead to improved results.

As demonstrated by the present study, GGOH addition antagonized the effect of Zol and, as a result, the activities of osteoblasts and osteoclasts were restored. However, the effect of GGOH was exerted on a limited number of genes. The Ras superfamily, influenced by FPPS, comprises various subfamilies, including Ras, Rho, Rab, Ran, and Arf [35], and the prenylation of the proteins is mediated by three enzymes, namely, farnesyltransferase, geranylgeranyltransferase-I, and geranylgeranyltransferase-II [27,30]; therefore, another type of isoprenoid, farnesol, may play an important role in the rescue of cellular functions. Additionally, the effect of GGOH was not sufficient to reach values demonstrated by the control. Different concentrations of GGOH may lead to the obtainment of better results, although the cytotoxic effect of GGOH was also observed at an extremely high concentration (80 μM) [27]. Furthermore, considering the well-established role of osteoblasts in regulating osteoclastogenesis, conducting a co-culture experiment involving osteoblasts and osteoclast progenitors under the influence of BPs and GGOH treatment, followed by the assessment of gene and protein expression, would provide valuable insights into the molecular mechanisms underlying the effects of BPs and GGOH on the interplay between osteoblasts and osteoclasts.

To manage the conditions associated with MRONJ, various adjuvant therapies, including hyperbaric oxygen (HBO) therapy, parathyroid hormone analog teriparatide treatment, and laser treatment, have been reported [30]. However, a fundamental approach at the cellular level has not been applied in clinical settings thus far. The results show a possibility that the topical application of GGOH in MRONJ patients along with surgical treatment, or, for preventive purposes, during dentoalveolar surgery in patients with bisphosphonates, might lessen the risk of development and recurrence of medication-related osteonecrosis of the jaw (MRONJ), since GGOH aids the recovery of cell viability and functions, resulting in improved bone turnover. Moreover, the anti-inflammatory and neuroprotective effects of GGOH have also been reported, in addition to antagonization of the effect of bisphosphonates [36]. However, preclinical and clinical studies are essential to verify the results and its possible use in clinical settings. Systemic administration may not be recommended because of the possible risk of compromising the purpose of bisphosphonate therapy.

## Figures and Tables

**Table 1:** Primer sequences used for RT-PCR.

Gene	Forward (5′-3′)	Reverse (5′-3′)
*ALP*	CCGTGGCAACTCT ATCTTTGG	GCCATACAGGAT GGCAGTGA
*OC*	AAGAGACCCAGG CGCTACCT	AACTCGTCACAG TCCGGATTG
*M-CSF*	CTCCAGAGAGAG GAGCCTGA	AGTATAGACACT CGTCACTGGTG
*RANKL*	ATACCCTGATGAAAGGAGGA	GGGGCTCAATCTATATCTCG
* COL1 *	CCCTGGAAAGAATGGAGATGAT	ACTGAAACCTCTGTGTCCCTTCA
*RUNX2*	AGCAAGGTTCAACGATCTGAGAT	TTTGTGAAGACGGTTATGGTCAA
* RANK *	TGTGGCACTGGATCAATGAG	GTCTTGCTGACCAATGAGAG
*TRAP*	GATCCTGGGTGCAGACTTCA	GCGCTTGGAGATCTTAGAGT
*CALCR*	GACAACTGCTGGCTGAGTG	GAAGCAGTAGATGGTCGCAA
*CSF1R*	GGCTCCTGGGCCTTCATACC	CAAAGGCTCCAGCTCCGAGG
* c-Fos *	TGTCTGTGGCTTCCCTTGAT	ATCAAAGGGCTCGGTCTTCA
* OSCAR *	GAGTAGCTGAAAGGAAGACGCGAT	CAGAGCGCTGATTGGTCCATCTTA

## Data Availability

Data supporting the reported results can be provided upon reasonable request.
